# Formation of a Conducting Polymer by Different Electrochemical Techniques and Their Effect on Obtaining an Immunosensor for Immunoglobulin G

**DOI:** 10.3390/polym15051168

**Published:** 2023-02-25

**Authors:** Erika Martinez-Sade, Francisco Martinez-Rojas, Danilo Ramos, Maria Jesus Aguirre, Francisco Armijo

**Affiliations:** 1 Departamento de Química Inorgánica, Facultad de Química y de Farmacia, Pontificia Universidad Católica de Chile, Avenida Vicuña Mackenna 4860, Macul, Santiago 7820436, Chile; 2 Millenium Institute on Green Ammonia as Energy Vector, Pontificia Universidad Católica de Chile, Santiago 7820436, Chile; 3 Departamento de Química de Los Materiales, Faculta de Química y Biología, Universidad de Santiago de Chile, USACH, Av. L.B. O’Higgins 3363, Santiago 9170022, Chile; 4 Centro de Nanotecnología y Materiales Avanzados, CIEN-UC, Pontificia Universidad Católica de Chile, Santiago 7820436, Chile

**Keywords:** nanostructure, conducting polymer, immunoglobulin G, electrochemical immunosensor, square wave voltammetry

## Abstract

In this work, a conducting polymer (CP) was obtained through three electrochemical procedures to study its effect on the development of an electrochemical immunosensor for the detection of immunoglobulin G (IgG-Ag) by square wave voltammetry (SWV). The glassy carbon electrode modified with poly indol-6-carboxylic acid (6-PICA) applied the cyclic voltammetry technique presented a more homogeneous size distribution of nanowires with greater adherence allowing the direct immobilization of the antibodies (IgG-Ab) to detect the biomarker IgG-Ag. Additionally, 6-PICA presents the most stable and reproducible electrochemical response used as an analytical signal for developing a label-free electrochemical immunosensor. The different steps in obtaining the electrochemical immunosensor were characterized by FESEM, FTIR, cyclic voltammetry, electrochemical impedance spectroscopy, and SWV. Optimal conditions to improve performance, stability, and reproducibility in the immunosensing platform were achieved. The prepared immunosensor has a linear detection range of 2.0–16.0 ng·mL^−1^ with a low detection limit of 0.8 ng·mL^−1^. The immunosensing platform performance depends on the orientation of the IgG-Ab, favoring the formation of the immuno-complex with an affinity constant (Ka) of 4.32 × 10^9^ M^−1^, which has great potential to be used as point of care testing (POCT) device for the rapid detection of biomarkers.

## 1. Introduction

The development of point-of-care testing (POCT) and rapid tests for the detection of antibodies in recent years, complemented by research in the field of human immune status, has made it possible to identify a group of diseases associated with impaired immunoglobulin G synthesis (IgG) [1,2]. The IgG is the major antibody in human serum, forming approximately 70% of total immunoglobulins. In addition, IgG consists of four subclasses: IgG1, IgG2, IgG3, and IgG4, presenting each of the IgG subclasses different concentrations in blood serum: IgG1—300 mg/dL; IgG2—50 mg/dL; IgG3—25 mg/dL and IgG4—1 mg/dL [3]. Therefore, the recognition of IgG in human serum has allowed the detection and treatment of numerous infectious diseases, such as botulism, syncytial virus, COVID-19, among others [4,5,6,7,8].

Recently, many immunoassay methods for the detection of IgG have been studied such as enzyme-linked immunosorbent assay (ELISA) [4], fluorescent immunoassay [5], capillary electrophoresis [6], photoelectrochemical immunoassay [7], surface-enhanced Raman scattering (SERS) [8], and so on. Although these methods have been developed for many years in detecting immunoglobulin antigens (IgG-Ag), they present some disadvantages that make them more expensive, such as the greater requirement of equipment and reagents, longer time for sample preparation, and need for highly qualified personnel. Therefore, there is still a need for the development of methods for detection to have better sensitivity and selectivity to achieve rapid and dynamic concentration response of IgG-Ag in real samples [9].

Consequently, as an alternative, the development of electrochemical immunosensors arises; these have advantages such as requiring very small sample volumes for the detection of an analyte (microliters), being easy to miniaturize, and their sensitivity is not affected during measurements. In addition, large-scale production of immunosensor electronic devices is low cost with excellent performance for complex samples [10,11,12,13,14,15,16].

The production of robust and reproducible bio-electrochemical platforms with high sensitivity requires the covalent immobilization of immunoglobulin antibodies (IgG-Ab) in a site-specific manner, which contributes to better performance, instead of the random IgG-Ab orientations resulting from the direct yet non-selective immobilization techniques [17,18,19,20]. Ramanaviciene et al. reported that the best antigen detection sensitivity was monitored using an SPR-chip modified with oriented antibody fragments obtained after reduction with dithiothreitol [18]. Vasile et al., to overcome the immobilization of antibodies onto hydrophobic polymeric surfaces with disordered orientation, controlled IgG-Ab immobilization onto poly(vinylidene fluoride) surface carry out, performing a two-step process involving radiofrequency plasma pretreatment for polymer surface functionalization, followed by coupling reaction via protein-A [19]. Different materials have also been reported for obtaining electrochemical platforms, and their covalent functionalization with antibodies (Abs) in an orderly and controlled way by means of a two-step process. The first step is the activation of the free carboxylic groups (-COOH) found in the suitable functionalized support in contact with an EDC/NHS solution, followed by the bioconjugation of the amino group found in the Abs through an amide-type covalent bond [17,20,21,22]. Among the electrochemical platforms reported for IgG-Ag detection are those that immobilize different bio receptors, including DNA-peptides, peptide aptamer, multifunctional peptide, and IgG-Ab. Additionally, the same platforms have used the following signal amplifiers: metal nanoparticles, polyaniline nanowire arrays, metal-organic frameworks, quantum dots, and carbon nanotubes [23,24,25,26,27,28,29,30].

On the other hand, the conducting polymers (CPs) poly(3,4-ethylenedioxythiophene) (PEDOT) [23,26] and polyaniline [24,25] were electrodeposited on the electrode surface to form an antifouling and conducting interface with three-dimensional pore structure allowing the immobilization of the bioreceptor to identify IgG. CPs can be used in different applications due to their physicochemical characteristics such as good conductivity, stability, mechanical flexibility, low toxicity, biocompatibility, and antifouling properties [31,32,33,34]. In addition, CPs with nanometric structures can be obtained at low cost, showing stable electrochemical responses due to the increased active area, which demonstrates their potential use in capacitors and electroanalytical sensors [32,33,34,35]. In recent years, interest in obtaining CPs from indole derivatives has increased, and studies of the effect of the position of a carboxylic group in different positions of the indole molecule indicated that the position of the substituent influenced the size and diameter of the nanowires, and therefore in the electrochemical properties [35]. Some electrochemical [21,22,36,37,38,39,40,41,42,43], electrochemiluminescence [44,45,46,47] and photoelectrochemical [48] biosensing platforms for the detection of different biomarkers have been reported, where polyindole derivatives and carboxylic acid form part of the modified electrode. However, there are no reports of studies on the response of an electrochemical immunosensor when the same conducting polymer is obtained electrochemically by different electrochemical techniques classified as galvanostatic, potentiostatic and potentiodynamic, and depending on which is applied, the CPs may present different physical-chemical properties.

Generally, when antigens and antibodies are not intrinsically electroactive, two classes of electrochemical immunosensors are used: indirect (label) and direct (label-free). Indirect immunosensors use different labels such as enzymes and electroactive compounds directly attached to biomolecules which allow them to follow a redox reaction as an analytical signal. The main disadvantages of these systems are the instability of the enzymes, the effect on the efficiency of the formation of the immunocomplex due to having a marker that can affect the conformation of the biomolecules, the need for very specific reagents, and additional procedures [49]. Faced with these disadvantages, direct immunosensors have received increasing interest due to their ability to detect the formation of the antigen-antibody immune complex, which changes the electrical properties of the system and can be followed directly by an electrochemical technique [49]. Therefore, the physical and chemical properties of the immunosensor platform should provide optimal conditions for antibody immobilization and avoid non-specific interactions of other interfering proteins and ions found in biological fluids, which may affect the electrochemical response.

This work reports the detection of antibody IgG using a label-free electrochemical immunosensing method. For the fabrication of this immunosensor, only the modification of a glassy carbon electrode with poly-indole-6-carboxylic acid (6-PICA/GCE) was necessary. The nanostructure obtained makes it possible to direct immobilization of the IgG antibody, and by means of the electrochemical response by square wave voltammetry allows it to follow the formation of the immune complex. This immunosensor shows low detection of limit, a stable electrochemical response, great sensitivity, and a remarkable absence of interferences present in biological samples, contributing to the clinical diagnosis of various diseases and derived applications in the biosensor field.

## 2. Materials and Methods

### 2.1. Materials and Reagents

All reagents used were of analytical grade without any previous treatment. The 6-indole-carboxylic acid (6-ICA), bovine serum albumin (BSA), prostate-specific antigen (PSA), carcinoembryonic antigen (CEACAM), human serum, IgG from human serum (IgG-Ag = 150 KDa), anti-human IgG antibody (Fab specific) produced in goat (IgG-Ab), N-(3-dimethylaminopropyl)-N′-ethylcarbodiimidehydrochloride (EDC), N-hydroxysulfosuccinimide sodium salt (NHS), lithium perchlorate (LiClO_4_), and other chemicals were purchased from Sigma-Aldrich. Proteasome 20S (human) purified (P20S) was purchased from ENZO life sciences. Acetonitrile (ACN) HPLC grade was purchased from PanReac and Applichem. Acetic buffer solution pH 4.75 and, all solutions were prepared with Milli-Q grade water (18 MΩ·cm^−1^) and stored at −20 °C.

### 2.2. Electrochemical and Spectroscopic Measurements

The electrochemical measurements were performed using a CHI-720 potentiostat and a three-compartment electrochemical cell. The glassy carbon working electrode (GCE; 0.125 cm^2^) was supplied by CH Instruments, Inc. (Texas, USA). A platinum wire was used as the auxiliary electrode, while Ag?AgCl?KClsat electrode was used as the reference electrode.

The electrochemical techniques used were:

- Cyclic voltammetry (CV) between −0.4 and +0.8 V at performed at different scan rates.
- Square wave voltammetry (SWV) The optimized parameters were: potential step 5 mV, amplitude 25 mV, and frequency 10 Hz, and the potential range was between −0.2 V and +0.65 V, for the initial and final potential, respectively.
- Electrochemical impedance spectroscopy (EIS) measurements were carried out to formal potential, E^0^’, which was determined from the average of the anodic and cathodic peak potentials, over a frequency range of 100 kHz to 0.01 Hz, at 8 step/decade, using a perturbation of 10 mV.

All electrochemical measurements were carried out in 1.0 mol·L^−1^ acetate buffer solution.

The morphological characterization of the resulting coatings on highly oriented pyrolytic graphite (HOPG) electrode was determined by FE-SEM (Quanta Feg 250 scanning electron microscope in a high vacuum mode under an acceleration voltage of 5 kV). FT-IR (ATR) measurements were carried out using Thermo Fisher Scientific Nicolet (Massachusetts, WA, USA) is −10 equipment.

### 2.3. Obtention of Immunosensor Based on 6-PICA

Conducting polymer 6-PICA was obtained on a glassy carbon electrode (GCE) from 10 × 10^−3^ mol·L^−1^ 6-ICA + 0.1 mol·L^−1^ LiClO_4_/ACN solution using three different electrochemical techniques:

Method 1: Cyclic voltammetry (CV) between 0 V and 1.0 V at 80 mVs^−1^ for 30 cycles.

Method 2: Pulse potential was carried out for 170 cycles at two potentials: the first one at oxidation potential 1.1V for 4 s and −0.3 V for 1 s.

Method 3: Chronoamperometric was carried out at 1.1 V and applied for 600 s.

The next step was the activation of carboxylic groups, which were activated with 20 mM EDC/NHS. Then, 50 μL of acetate buffer (pH = 4.75) containing 50 μg mL^−1^ IgG was dropped onto the surface 6-PICA/GCE and incubated for 2 h, which was then incubated again in 10 μL of 2% BSA for another 1 h to block the remaining active sites and avoid nonspecific adsorption. Finally, IgG-Ab/6-PICA/GCE electrodes were incubated at different concentrations of IgG-Ag for 1 h. All measurements and incubation steps were achieved at room temperature and with acetate buffer (pH = 4.75).

### 2.4. Analytical Procedure

IgG detection was carried out by SWV, the IgG-Ab/6-PICA/GCE electrode was incubated with different IgG-Ag concentrations between 2 and 16 ng·mL^−1^ from a stock solution of 200 ng·mL^−1^ for 1 h at room temperature under continuous stirring. Analytical parameters, such as linear range, limits of detection (LOD), and quantification (LOQ) were determined.

### 2.5. Optimization of Anti-IgG Antibody and IgG Antigen Incubation

The optimizations of IgG-Ab and IgG-Ag were focused on incubation time. First, 6-PICA/GCE electrode was incubated in 50.0 μL of IgG-Ab solution of 50.0 µg·mL^−1^ for 2, 4, 6 and 24 h. IgG-Ag/6-PICA/GCE electrode was incubated in 50.0 μL of IgG-Ag solution of 100.0 ng·mL^−1^. Both optimizations were achieved in acetate buffer (pH 4.75) and voltammetric responses were recorded by CV and SWV.

### 2.6. Human Serum Analysis

Human serum samples were reconstituted in acetate buffer (pH 4.75) for a final concentration of IgG-Ag 6 ng·mL^–1^ and stored at −20 °C. Electrochemical detection was performed by SWV using the standard calibration addition method, adding six standard incubations of 2 ng·mL^–1^ each one, over the same electrode.

## 3. Results

### 3.1. Morphological and Electrochemical Characterization of 6-PICA Platform

**Figure 1:**
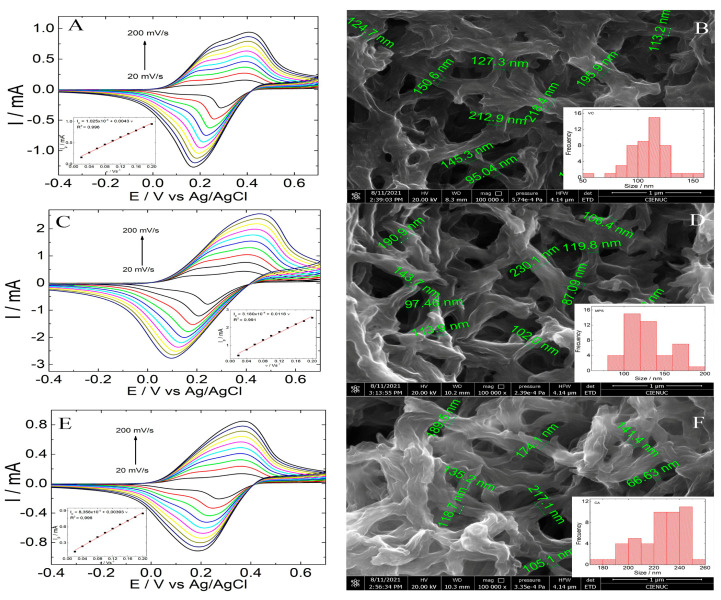
CV (**A**,**C**,**E**) in acetate buffer 0.1 mol·L^−1^ (pH 4.75) at different scan rates of 0.2 V·s^−1^ to 0.02 V·s^−1^ and SEM images (**B**,**D**,**F**) of 6-PICA obtained through three different electrochemical techniques. Inset (**A**,**C**,**E**): the linear relationships of the anodic peak current depended on different scan rates. Inset (**B**,**D**,**F**): histogram showing the nanowire size distributions of 6-PICA.

Figure 1A–F shows the scan rate study by CV and SEM images of 6-PICA/GCE obtained through three different electrochemical techniques: CV (Figure 1A,B), pulse potential (PP) (Figure 1C,D), and chronoamperometry (Chr) (Figure 1E,F). An utter nanostructure deposited over the entire GCE surface homogeneous covering in all was observed. The images show a magnification of 100,000×, where a nanowire-type structure can be seen with different diameter sizes interconnected in a fibrous network; these morphologies are similar to those reported in other research [21,22,35,36,44]. Inset: Figure 1B,D,F the histograms obtained are shown, can be seen different size distribution that depends on the electrochemical technique used. The materials obtained by CV present the most homogeneous distribution between 100 and 125 nm, the other two techniques present a greater distribution of sizes. Figure 1A,C,E show in the potential range of −0.4 V to 0.8 V a stable current response in each scan rate of 20 to 200 mV·s^−1^. The 6-PICA film obtained by CV, PP, and Chr exhibits two well-defined redox couples, indicating reversible and fast electron transfer processes. The anodic peak currents (I_p_) vs. scan rate (ν) plots in inset Figure 1A,C,E exhibit a linear trend; the fact that the currents are linearly correlated with the scan rate suggests that a surface covered with an electroactive film exhibits electrochemical response characteristics of redox species confined on substrate electrode. The peak current (*I_p_*) for an adsorbed compound allows for determining apparent surface coverage (*Γ*) by the Equation (1) [21]:

(1) $$I_{p}=n^{2}\;F^{2}\;\nu \;A\;\Gamma \;/\;4RT$$

where *A* is the area of the electrode surface (0.125 cm^2^); *n* is the number of electron transfers; *F* is Faraday constant (96 485 C·mol^−1^); *R* is gas constant (8.314 J·mol^−1^ K^−1^); and *T* is the room temperature (298.15 K). Assuming a one-electron process, the (*Γ*) calculated were 5.19 × 10^−8^ mol/cm^2^ for VC, 3.61 × 10^−8^ mol/cm^2^ for Chr, and 1.25 × 10^−7^ mol/cm^2^ for PP.

Although the large specific area provided by this 3D structure shape of the 6-PICA allows direct immobilization of the antibody, only the modified electrode obtained through CV shows a stable and reproducible electrochemical response after bioconjugation. Moreover, one of the drawbacks observed is the lower adhesion of the conducting polymer on the glassy carbon when 6-PICA was obtained by PP and Chr. Additionally, it offers a suitable bioplatform/electrolyte interface for the doping-undoping process, which results in an electrochemical response as an analytical signal [21,22]. Therefore, the 6-PICA nanostructure obtained by CV presents the best electrochemical characteristics to develop an electrochemical immunosensor for a direct detection test.

### 3.2. Label-Free Electrochemical Immunosensor Fabrication

**Figure 2:**
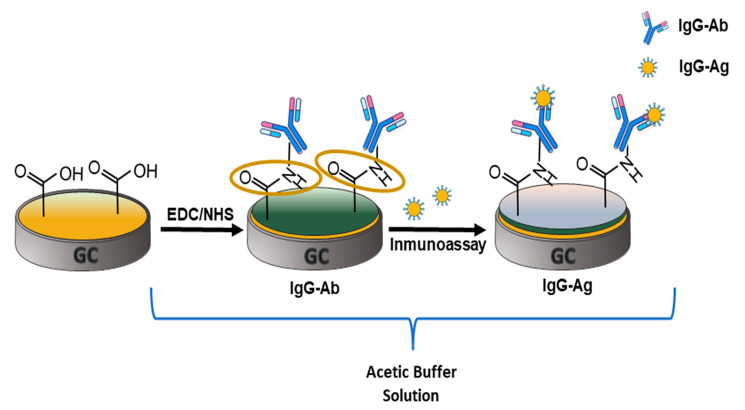
Schematic fabrication protocol of the electrochemical immunosensor for the detection of IgG-Ag.

An illustration of the three steps required to obtain the direct electrochemical immunosensor is shown in Figure 2. Obtaining 6-PICA/GCE under CV optimal conditions is the initial stage, then the modified electrode is subjected to potentiodynamic cycles until obtaining an electrochemical response that is consistent and repeatable in an acetate buffer (pH 4.75). The conducting polymer becomes unstable at higher pHs and separates from the electrode surface. Due to this, the subsequent procedures were carried out at this pH to enable IgG-Ag detection. In the second stage, the 6-PICA’s free carboxylic groups (-COOH) are activated by coming into contact with an EDC/NHS solution. Next, the IgG-Ab amino group is directly bioconjugated by an amide-type covalent link, resulting in IgG-Ab/6-PICA/GCE. The incubation of IgG-Ag on IgG-Ab/6-PICA/GCE is the third stage. The function of the immunosensor depends on the orientation of the antibody, and this process enables directed immobilization of IgG-Ag, favoring the creation of the IgG-Ag-Ab-IgG immunocomplex. All these procedures are crucial to the development of the immunosensing platform [17,18].

**Figure 3:**
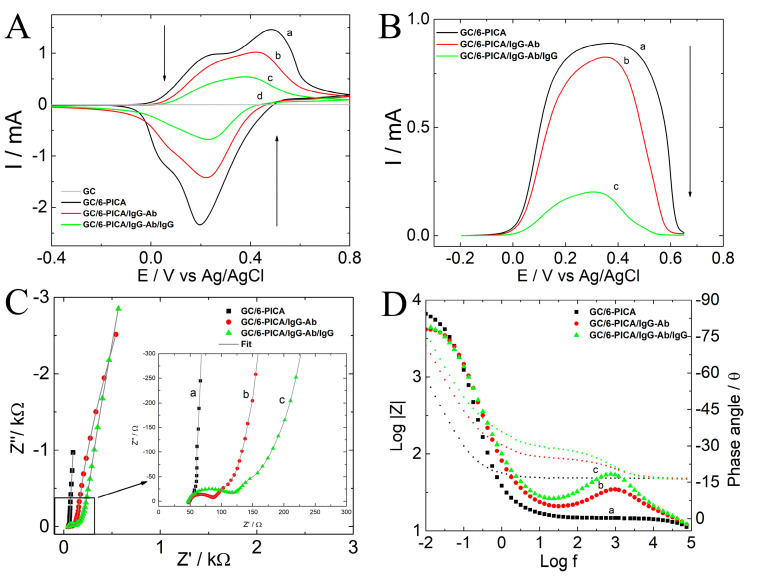
(**A**) CV. Potential scan rate: 0.1 V·s^−1^. (**B**) SWV to the optimized parameters. (**C**) Nyquist and (**D**) Bode plots. EIS: to formal potential, E^0^, 0.01 Hz to 100 KHz, 10 mV of the amplitude of (a) 6-PICA/GCE (black squares), (b) IgG-Ab-6-PICA/GCE (red circles), (c) IgG-Ag-Ab-IgG-Ab-6-PICA/GCE (green triangles), (the concentration of IgG-Ag is 100 ng·mL^−1^) in acetate buffer (pH 4.75), (d) GCE.

Figure 3A–D shows the electrochemical responses obtained by CV, SWV, and EIS in acetate buffer (pH 4.75) step by step in the fabrication of the sensing interface for IgG detection.

Figure 3A,B shows voltammetric profiles for 6-PICA/GCE (black line), IgG-Ab/6-PICA/GCE (red line), and IgG-Ag/IgG-Ab/6-PICA/GCE (green line), respectively. In both figures a reduction in the peak current occurs once the IgG-Ab/6-PICA/GCE system is formed, then another decrease in the peak current occurs in the recognition process responsible for the detection of the antigen (IgG -Ag/IgG-Ab/6-PICA/GC). The CV and SWV demonstrated clear changes in electrochemical responses, following each modification step and particularly during the detection stage (c), the charge transfer between the conducting polymer and the solution decreases due to the presence of antibodies and immobilization of the antigen, both non-conductors, supplying the analytical signal required to determine IgG-Ag. Since the peak current achieved in this study is proportional to the amount of IgG-Ag in the sample, SWV was chosen as the electroanalytical detection technique.

After each modification procedure, the electrode surface was also characterized using EIS. The quality of the fitting was evaluated by an acceptable error value χ^2^ < 0.001 (Table 1). One recent study showed that it is possible to fit modeling the lack of homogeneity on the surface of a modified electrode using a modified equivalent Randles circuit in which a constant phase element (CPE) replaces the double-layer capacitance (Cdl) [21,22]. To sense changes in the electrical characteristics at the electrode/solution interface, certain electrochemical biosensors use a redox probe in the solution [23,26]. The modified electrode can be affected and harmed by these redox probes, which can also produce corrosion on substrate electrodes and deposit on their surface. The effects of each of these processes on the analytical signal utilized for detection make it difficult to identify the target molecule quantitatively. Other investigations employed an analytical signal linked to the faradaic response of redox groups directly bonded to the electrode surface in an effort to address these drawbacks [37,38,39]. Accordingly, because it depends on changes in the nano-environment on the electrode surface during the modification steps, the intrinsic redox response of the 6-PICA corresponding to the doping-undoping processes was chosen as an analytical signal in this study.

Figure 3C,D show the Nyquist and the Bode plots for each step in obtaining the electrochemical immunosensor. The general Randles circuit includes the ohmic resistance (Rs = R1) of the electrolyte solution, the double layer capacitance (Cdl = CPE1), the electron transfer resistance (Rct = R2), and Warburg impedance (Zw = CPE2) resulting from the diffusion of ions from the bulk electrolyte to the interface electrode/solution. Data fitting for the 6-PICA/GCE was performed using the Randles model, but for the IgG-Ab/6-PICA/GCE and the IgG-Ag/IgG-Ab/6-PICA/GCE, the impedance data were fitted using a circuit that introduces a relaxation time constant (τ_0_), adding resistance R3 and the CPE3 in series between the R1 and the Randles circuit used for the 6-PICA/GCE (Table 1). Each modified electrode exhibits a negligible change in the resistance of the solution (R1) (Table 1), which is consistent with a constant electrolyte concentration. R2 controls the electron transfer kinetics of the interface electrode/solution. It is a suitable parameter for sensing the interfacial properties of the modified electrodes. In Figure 3C, the semicircle diameter of EIS equals the electron transfer resistance. R2 increases (Table 1) when IgG-Ab modifies 6-PICA/GCE (red circles) and IgG-Ag is bound by its specific antibody (green triangles). In Figure 3D, at high frequencies, a relaxation time constant (τ_0_ = R3-CPE3) was observed for IgG-Ab/6-PICA/GCE and IgG-Ag/IgG-Ab/6-PICA/GCE, a τ_0_ was determined to be 1.02 and 1.33 ms, respectively. This suggests that the modified electrode has a different active site for charge storage generation. The fact that the antibody and the antigen were successively immobilized on the surface of 6-PICA, generating layer by layer an insulator capable of obstructing the transfer of charge between interfaces, also supports the increase in resistance values found for R2 and R3 (Table 1). Therefore, EIS is a powerful tool for the characterization of these electrochemical systems, and the relative change of the electron transfer resistance can be monitored as a function of the antigen concentration.

**Figure 4:**
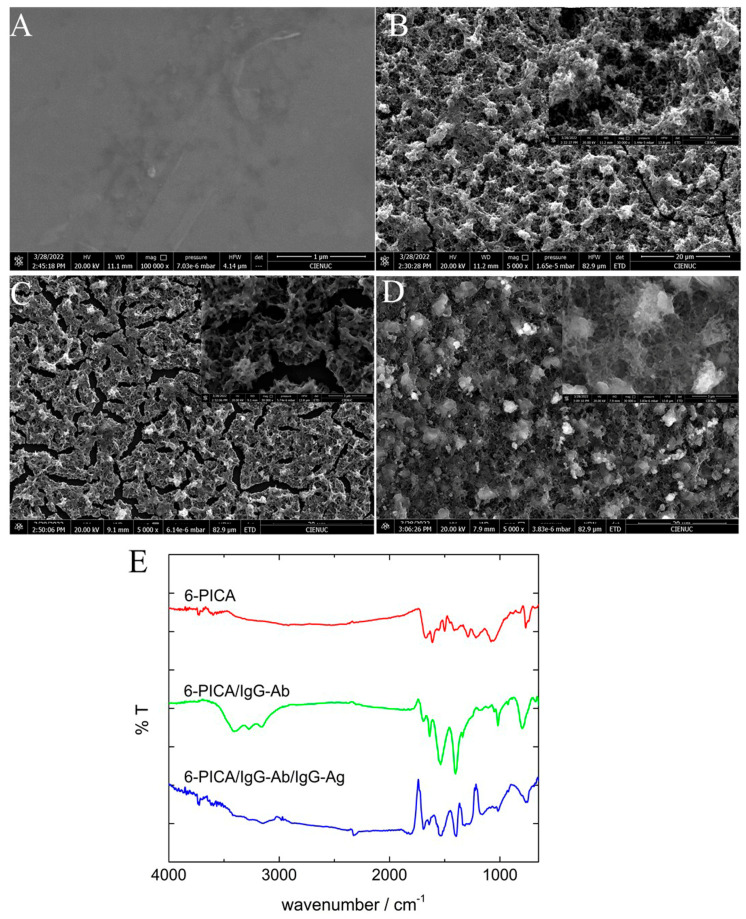
SEM images of (**A**) HOPG, (**B**) 6-PICA/HOPG, (**C**) IgG-Ab/6-PICA/HOPG before and after (**D**) the addition of the antigen (IGg-Ag/IgG-Ab/6-PICA/HOPG) on 5000×, the scale bars represent 20 μm. Inset. The scale bars represent 3 μm. (**E**) FT-IR spectra of the 6-PICA (red line), IgG-Ab/6-PICA (green line), and IGg-Ag/IgG-Ab/6-PICA (blue line).

The morphology of the HOPG, 6-PICA/HOPG, IgG-Ab/6-PICA/HOPG, and IgG-Ag/IgG-Ab/6-PICA/HOPG electrode was analyzed using SEM to verify that the biorecognition reaction was successfully carried out. The unmodified bare electrode exhibits a smooth structure (Figure 4A), and the 6-PICA electrode shows a rough surface with a uniform distribution of different-sized nanostructures (Figure 4B). In Figure 4C, after bioconjugation it was observed that the antibody molecules look like bright particles with a general size of 1–2 µm on a cracked surface that have irregular shapes. After reacting with the antigen (Figure 4D), the particles grow bigger (2–3 µm) and present a greater agglomeration of irregular shapes due to the lower conductivity. The difference between the two interfaces indicates the formation of the immune complex, indicating the feasibility of applying the direct immunosensor.

On the other hand, Figure 4E shows the transmission FT-IR spectra in ATR mode for 6-PICA (red line), IgG-Ab/6-PICA (green line), and IgG-Ag/IgG-Ab/6-PICA (blue line). The band of 6-PICA at 1672 cm^−1^ was related to the stretching vibration of the C=O group, the band at 1213 cm^−1^ was assigned to band C−O stretching, the band at 1070 cm^−1^ attributed to the vibrational modes of the dopant ClO_4_. The N-H bond’s elongation and deformation vibrations are responsible for weak and wide bands between 3140 and 3410 cm^−1^ and the band at roughly 1555 cm^−1^. The bands associated with the oxidized 6-PICA are also in the spectral region between 3540 and 3660 cm^−1^, which is a property of hydroxyl groups. Similar results have been reported elsewhere [21,35]. The FT-IR spectra for IgG-Ab/6-PICA show peaks of amide I, II, and A bands or peptide characteristic peaks [50,51,52], three bands at 3143, 3276, and 3399 cm^−1^ from the N-H stretching vibration, two bands at 1635 and 1688 cm^−1^ correspond to the stretching mode of C=O, and at 1544 cm^−1^ from the bending mode vibration of N-H in the amide group, respectively. In addition, the FT-IR spectrum of the IgG-Ag/IgG-Ab/6-PICA when the antigen recognition is performed shows the disappearance of several signals, maintaining the amide II band at 1548 cm^−1^ and the amide I band at 1696 cm^−1^. Consequently, the spectroscopic data show the immobilization of the antibody on the conducting polymer and the formation of an immunocomplex that allows the corresponding detection of the antigen.

### 3.3. Optimization of Method Label-Free Electrochemical Immunosensor

**Figure 5:**
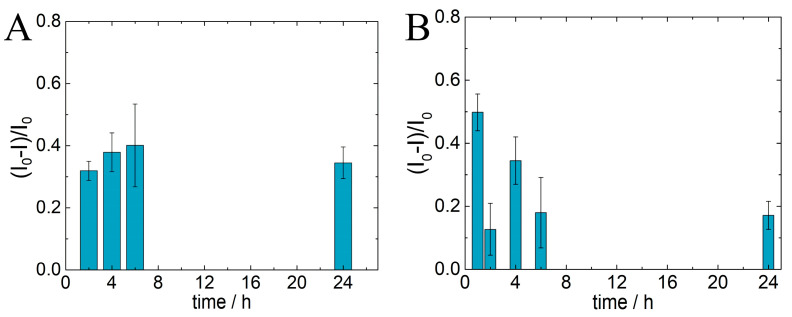
SWV response optimization of the performance of the immunosensor in acetate buffer 1.0 mol·L^−1^ (pH 4.75). (**A**) incubation time of IgG-Ab on 6-PICA/GCE. (**B**) incubation time of IgG-Ag on IgG-Ab/6-PICA/GCE.

IgG-Ab immobilization on 6-PICA/GCE and IgG-Ag recognition were optimized for incubation times between 1 and 24 h in Figure 5A,B, respectively. The plots were produced from the optimal voltammetric response obtained by SWV using the analytical ratio of the decreased peak currents, ΔI = (I_0_ − I)/I_0_ [21], where (I_0_) is the peak current of 6-PICA/GCE and IgG-Ab/6-PICA/GCE, respectively, and (I) is the peak current following the binding of IgG-Ab to 6-PICA (Figure 5A) and IgG-Ag to IgG-Ab/6-PICA/GCE (Figure 5B). AgG-Ab on 6-PICA/GCE incubation adjustments for times between 2 and 24 h are shown in Figure 5A. Given that the decrease in the current peak has a great effect on the analytical response of the immunosensor, an increase can be observed for the standard deviation (RSD) of ΔI at six hours and a similar value can be observed between 1 and 24 h. A lower RSD in the measurements taken is very important since it tells us how reproducible the immobilizations were achieved. To test for the interaction of IgG-Ag on the immunosensing platform, Figure 5B shows the optimization of the incubation time of IgG-Ag on IgG-Ab/6-PICA/GCE at 1, 2, 4, 6, and 24 h. A significant fluctuation in ΔI was seen at various times, but with a low RSD for ΔI at a shorter incubation time. It is always advisable to obtain the analytical response in the shortest possible time, thus the parameters with the lowest RSD for ΔI were chosen in order to obtain the best experimental conditions. Overall, it was established that 2 h were optimal for immobilizing IgG-Ab on the 6-PICA while 1 h was best for IgG-Ag detection.

### 3.4. Quantitative Detection of IgG-Ag

**Figure 6:**
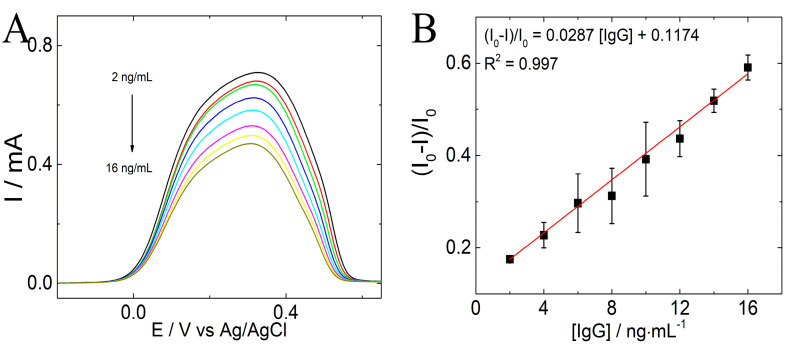
(**A**) SWV of IgG-Ab/6-PICA/GCE in acetate buffer 1.0 mol·L ^−1^ (pH 4.75) in the presence of different concentrations of IgG-Ag among 2 to 16 ng·mL^−1^. (**B**) Calibration curve for IgG-Ag detection. Plot ΔI vs. [IgG-Ag] ng·mL^−1^.

The electroanalytical measurements of the immunosensor were performed under optimal conditions in an acetate buffer (pH 4.75) by SWV. Figure 6A shows the SWV response of biosensor devices in the presence IgG-Ag with concentrations ranging from 2.0 to 16.0 ng·mL^−1^; as concentrations increase, the peak current decreases, due to the immunocomplex formation. The electrochemical immunosensor can be used only once for each calibration curve and then must be discarded.

Figure 6B demonstrates the observed ΔI with respect to IgG-Ag concentration. The immunosensing platform shows an acceptable linear correlation (r^2^ = 0.997) and a limit of detection (LOD) and quantification (LOQ) of 0.8 and 1.8 ng·mL^−1^, respectively (*n* = 3) was obtained. LOD and LOQ were calculated by the 3σ method [21,22].

Table 2 compares a number of previously described electrochemical immunosensors [23,24,25,26,27,28,29,30]. Some of these biosensors are based on the use of specific peptides to recognize IgG, but they must be immobilized on complex platforms to show high specificity and selectivity, with a signal amplification strategy using nanostructures of different materials [23,24,25,26]. Additionally, generating molecularly imprinted electrochemical biosensors necessitates a number of operational processes and washing procedures before use, which hinders repeatability and restricts their suitability for point-of-care analysis [27,28,29,30]. Although these present linear range and LD acceptable will be costlier and more complicated to obtain. Therefore, IgG-Ab/6-PICA/GCE meets the goal of the urgent need to develop single-step and label-free assays for IgG analysis, with analytical parameters that allow the detection of IgG at clinically relevant concentrations.

### 3.5. Selectivity, Stability, and Affinity Constant of the Immunosensor and Serum Sample Analysis

To assess the selectivity of the immunosensing platform and ensure that the change in the current is due to antigen binding and not the settling of proteins on the surface, a test of some compounds that may be in negative controls of real samples was conducted, including bovine serum albumin (BSA), carcinoembryonic antigen (CEA-CAM), prostate-specific antigen (PSA), 20S proteasome, ascorbic acid (AA), glucose, and dopamine. The trials show no significant change in ΔI, indicating that the developed immunosensor has suitable selectivity for IgG-Ag detection (data not shown). Additionally, the proposed immunosensor’s stability was evaluated. The ΔI response was measured after IgG-Ab/6-PICA/GCE was stored at 4 °C in acetate buffer (pH 4.75) for 15 days in absence of IgG-Ag, the original signal decreased to 95.3%, indicating the immunosensor’s satisfactory stability. To ensure correct selectivity among IgG-Ag and other proteins that might coexist with IgG in biological samples, the high-affinity constant was obtained. The affinity constant (K_a_) of the immobilized antibody on 6-PICA and its antigen can be estimated from the graph of (I_0_ − I)/I_0_ vs. concentration [IgG-Ag] in molL^−1^, in accordance with the Langmuir isotherm model [53]. The affinity constant of monoclonal IgG-Ab for IgG-Ag is 4.34 10^9^ M^−1^. These analytical parameters are due to the stability of the electrochemical response of 6-PICA, showing similar behavior to immunosensors reported for other biomarkers [21,22].

For evaluating the sensor practicability, IgG-Ag was tested using a standard addition technique. Three equal amounts of spiked concentrations (6 ng·mL^−1^) were added to the human serum samples for the recovery experiment for IgG-Ag. According to Table 3, IgG-Ag recovery rate in serum samples of the immunosensor obtained by calculation and analysis was 106.0%, and the RSD was less than 2.92%. It showed that there was no significant cross-reaction between the components of the serum and the immunosensor, the biosensor constructed in the present study had sufficient accuracy in detecting serum IgG-Ag in serum samples.

The direct electrochemical immunosensor’s good performance is also attributable to its optimal biocompatibility, which enables the antibody to maintain its biological activity, and its conductivity, which enables a rapid charge transfer as a result of the doping-undoping process and can be used as an analytical signal. The performance of the immunodetection platform is being developed on screen-printed electrodes to be used as a point-of-care testing (POCT) device for the rapid detection of Ig-G and other biomarkers.

## 4. Conclusions

A direct electrochemical immunosensor was developed based on a nanostructured conducting polymer for the detection of IgG-Ag. The glassy carbon electrode modified with poly indol-6-carboxylic acid (6-PICA/GC) applied the cyclic voltammetry technique presented a more homogeneous size distribution of nanowires with greater adherence allowing the direct immobilization of the antibodies (IgG-Ab) to detect the biomarker IgG-Ag. In addition, 6-PICA presents the most stable and reproducible electrochemical response used as an analytical signal. Optimal conditions to improve the performance, stability and reproducibility of the immunosensing platform were achieved, selecting 2 h to immobilize IgG-Ab and 1 h for the detection of IgG-Ag to pH 4.75. Quantitative detection of IgG-Ag was carried out by SWV in a linear concentration range of 2.0 ng·mL^−1^ to 16.0 ng·mL^−1^, with a LOD of 0.8 ng·mL^−1^. In addition, a high IgG-Ag/IgG-Ab affinity constant, Ka, of 4.32 × 10^9^ M^−1^ was determined, therefore, providing an excellent sensing platform start for developing point-of-care test (POCT) devices for clinical diagnostics and other biosensor applications.

## Figures and Tables

**Table 1:** Fitting results of EIS data. The quality of the fitting to the equivalent circuit was evaluated by an acceptable error value of χ^2^ < 0.001. The changes in the electrochemical properties of the system were simulated using the modified Randles equivalent circuit model.

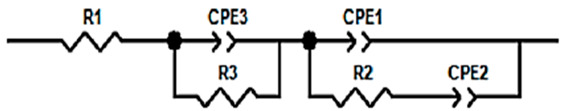			
**Electrode**	**6-PICA**	**6-PICA/IgG-Ab**	**6-PICA/IgG-Ab/IgG-Ag**
R1 (Ω)	49	48	46
CPE1-T (mF)	7.11	5.87	4.41
CPE1-P	0.97	0.69	0.55
R2 (Ω)	28	49	225
CPE2-T (mF)	7.22	0.0347	0.0288
CPE2-P	0.95	0.80	0.80
R3 (Ω)	-	38	64
CPE3-T (mF)	-	4.78	3.59
CPE3-P	-	0.92	0.95
χ^2^	3.33 × 10^−4^	3.20 × 10^−4^	4.21 × 10^−4^

**Table 2:** Comparison of different electrochemical immunosensors for the detection of IgG-Ag.

Electrode	Analytical Probe	Electrochemical Method	Lineal Range ng·mL^−1^	LOD ng·mL^−1^	Ref.
DP/AuNPs/PEDOT/GCE	[Fe(CN)_6_]^3−/4−^	DPV	0.1–10,000	0.037	[23]
Pep/BSA/PANI-NW/GCE	Direct detection	DPV	1.0–10,000	0.27	[24]
Pep/PANI/GCE	Direct detection	DPV	1.0–10,000	0.26	[25]
Y-peptide/AuNPs/PEDOT/GCE	[Fe(CN)_6_]^3−/4−^	DPV	0.1–10,000	0.032	[26]
NIH/GCE	[Fe(CN)_6_]^3−/4−^	EIS	0.5–200	0.03	[27]
MIP/CS/Cu-MOF/GCE	[Fe(CN)_6_]^3−^	DPV	0.01–10	0.003	[28]
MoS_2_@N-GQDs-IL MIP Sensors	[Fe(CN)_6_]^3−^	DPV	0.1–50	0.02	[29]
DTMI electrochemical biosensor	Direct detection	DPV	0.05–500	0.0288	[30]
IgG-Ab/6-PICA/GCE	Direct detection	SWV	2.0–16.0	0.8	This work

**GCE** = Glassy carbon electrode; **DP** = DNA-peptide conjugates; **AuNPS** = Gold nanoparticles; **PEDOT** = Poly(3,4-ethylenedioxythiophene); Pep = **peptide**; **PANI-NW** = Polyaniline; **NW** = Nanowires; **BSA** = Bovine Serum Albumine; **NIH** = Protein imprinted hydrogels; **Cu-MOF** = Copper metal-organic frameworks; **CS** = Chitosan; **MIP** = Molecularly Imprinted Polymers; **N-GQDs-IL** = Nitrogen doped graphene quantum dots–ionic liquid; **MWCNTs** = multi-walled carbon nanotubes; **DTMI** = Dual-template molecularly imprinted; **DPV** = pulse differential voltammetry.

**Table 3:** Concentration of IgG-Ag in human serum measured by SWV.

Sample	Added Spiked (ng·mL^−1^)	Found (ng·mL^−1^)	Recovery %
1	6	6.55	109
2	6	6.22	104
3	6	6.24	104

## Data Availability

Not applicable.
